# Modeling and Roles of Meteorological Factors in Outbreaks of Highly Pathogenic Avian Influenza H5N1

**DOI:** 10.1371/journal.pone.0098471

**Published:** 2014-06-02

**Authors:** Paritosh K. Biswas, Md. Zohorul Islam, Nitish C. Debnath, Mat Yamage

**Affiliations:** 1 Department of Microbiology, Chittagong Veterinary and Animal Sciences University, Chittagong, Bangladesh; 2 Food and Agriculture Organization of the United Nation, Dhaka, Bangladesh; National Institutes of Health, United States of America

## Abstract

The highly pathogenic avian influenza A virus subtype H5N1 (HPAI H5N1) is a deadly zoonotic pathogen. Its persistence in poultry in several countries is a potential threat: a mutant or genetically reassorted progenitor might cause a human pandemic. Its world-wide eradication from poultry is important to protect public health. The global trend of outbreaks of influenza attributable to HPAI H5N1 shows a clear seasonality. Meteorological factors might be associated with such trend but have not been studied. For the first time, we analyze the role of meteorological factors in the occurrences of HPAI outbreaks in Bangladesh. We employed autoregressive integrated moving average (ARIMA) and multiplicative seasonal autoregressive integrated moving average (SARIMA) to assess the roles of different meteorological factors in outbreaks of HPAI. Outbreaks were modeled best when multiplicative seasonality was incorporated. Incorporation of any meteorological variable(s) as inputs did not improve the performance of any multivariable models, but relative humidity (RH) was a significant covariate in several ARIMA and SARIMA models with different autoregressive and moving average orders. The variable cloud cover was also a significant covariate in two SARIMA models, but air temperature along with RH might be a predictor when moving average (MA) order at lag 1 month is considered.

## Introduction

The circulation of highly pathogenic avian influenza A virus subtype H5N1 (hereafter, HPAI H5N1) in several countries has been a serious global public health threat since 2003 because of its potential to mutate to initiate a human pandemic [1]–[3]. Apart from this, the subtype is zoonotic, already caused 377 deaths in 633 human clinical cases recorded in 15 countries, a case fatality rate of ∼60% [4]. To protect the public from the virus, and to stop a future human pandemic attributable to an HPAI H5N1 progenitor virus, the ideal is to eradicate the virus from poultry irrespective of geographical boundaries.

HPAI H5N1 has been detected in poultry and/or wild birds in 63 countries since 2003 when its international spread gained momentum [5], although the virus was first detected in diseased geese in the Guangdong province of China in 1996 [6]. It was reported in Bangladesh in 2007 [7], [8]. Most infected countries have eradicated it, but some resource-limited ones, particularly in South-East and South Asia [9] which have similar poultry rearing and geo-climatic factors, have not succeeded in doing so. HPAI outbreaks were predominantly clustered in three Asian countries - Bangladesh, Indonesia and Vietnam and in Egypt in the Middle East [9], [10]. Seasonal peaks have occurred in these and other countries [8], [10]–[14]. Factors contributing to such peaks need to be analyzed in order to forecast future events and to suggest interventions. Unnoticed reservoirs [15] and wild birds and their migrations [14], [16] have been blamed, but the roles of meteorological factors have never been studied. Modeling of climatological parameters on human influenza revealed some key meteorological factors contributing to temporal intensification and spread [17]–[19]. Here, for the first time, we illustrate the models and describe the roles of major meteorological factors in the occurrence of HPAI outbreaks in Bangladesh.

## Materials and Methods

### Climate and Seasons of Bangladesh

Bangladesh is between latitude 20°34′N and 26°38′N and between longitude 88°01′E and 92°41′E (http://www.banglapedia.org/HT/C_0288.HTM). It has a tropical monsoon climate. Except the hilly southeast, most of the country is a low-lying plain with a network of rivers and canals. Three distinct seasons can be recognized - the cool dry winter from November through February with January the coolest month, the pre-monsoon hot summer from March through May with April the hottest month, and the rainy monsoon, from June through October. March may also be considered as spring, and mid-October through mid-November may be called autumn (http://www.banglapedia.org/HT/C_0288.HTM).

### HPAI Outbreak Data

Bangladesh predominantly relies on passive reporting of HPAI outbreaks, based on farmers’ complaints of high mortalities in their flocks to the department of livestock services (DLS). Recently active surveillance has been introduced in 306 (62%) of the 482 upazilas (sub-districts) with support from Food and Agriculture Organization (FAO) of the United Nations, by its Avian Influenza Technical Unit (AITU). AITU has been working in close conjunction with the Epidemiology Unit of DLS. Based on the passive surveillance via DLS, and active surveillance via AITU, 550 HPAI outbreaks were confirmed from 2007 to April 2012. Their detailed data including dates of confirmation were available at AITU. Confirmation of HPAI was made by the National Reference Laboratory for Avian Influenza (NRL-AI), Dhaka, Bangladesh, by identifying M and H5 genes of the virus either by conventional or real-time reverse transcription polymerase chain reaction (RT-PCR) with extracted RNA from tracheal samples of dead birds referred to the laboratory. The confirmation of a HPAI outbreak was not based on the numbers of dead or sick chickens on a particular farm or from a defined area. When tracheal samples from dead chickens of any poultry farm sent to NRL-AI were diagnosed positive for the presence of H5 gene of the virus it was considered an outbreak. We collected epidemiological data of the 550 HPAI outbreaks in Bangladesh by April 2012, which are stored at AITU. The dates of confirmation of 529 HPAI outbreaks from 2007 through 2011 were the temporal records of interest for this study. Not having consistent information of all the meteorological variables for 2012 we excluded 21 outbreaks recorded in the first four months of 2012. Monthly numbers of HPAI outbreaks were aggregated for 60 months, from January 2007 to December 2011.

### Meteorological Data

Meteorological data were supplied from the Bangladesh Meteorological Department (Abhawa Bhaban, Agargaon, Dhaka, Bangladesh). Daily averages of the following were used: air temperature (°C) (TE), relative humidity (%) (RH), cloud cover (hour) (CC), rainfall (mm) (RF) and wind speed (knots) (WS) from 2007 to 2011. There are 35 meteorological observatory stations across the country to record daily data of different meteorological variables. Our meteorological data set contained separate daily average (not minimum and maximum ranges) records of the mentioned variables from each of these 35 stations. Daily solar radiation (Cal/cm^2^/min) is also recorded from these stations. The meteorological data that were provided from the Meteorological Department had missing records of daily solar radiation for the months August through December, 2007. Because of these missing data we finally dropped this variable from the analysis.

### Analysis

Daily average records of all the meteorological variables from each of the 35 observatory stations were entered into a spread sheet program (Microsoft Excel, 2007). A particular day’s arithmetic mean of a variable was calculated from the values of the day from all the 35 observatory stations. Aggregated daily means of a variable for a particular month was calculated. The number of outbreaks for a month was based on the date of confirmation of each outbreak.

A 3-month rolling mean of HPAI outbreaks was calculated from January 2007, and plotted as a bar chart to show the HPAI dynamics.

The HPAI time series analyzed in this study is characterized by autocorrelation. In order to account for the autocorrelation, a time series technique called Auto Regressive Integrated Moving Average (ARIMA) was employed [17], [20]. An ARIMA model is notated as ARIMA (*p,d,q*), where *p* indicates the autoregressive (AR) order, *d* the differencing order and *q* the moving average (MA) order. To compare seasonality of HPAI outbreaks we ran multiplicative seasonal auto-regressive integrated moving average (SARIMA) models with the same meteorological variables. A multiplicative seasonal SARIMA model is designated as SARIMA (*p, d, q*) (*P,D,Q*)_S_, where *p* and *P* indicate the autoregressive and seasonal autoregressive order, *d* and *D* the non-seasonal differences and seasonal differences and *q* and *Q* the moving average parameters and seasonal moving average parameters, respectively, and s represents the seasonal period. In this study the seasonal period was 12.

Cross-correlation function (CCF) between a meteorological variable series and the HPAI outbreak series was calculated to identify the lags to be included in the model. The significance of the cross-correlations was estimated on the basis of P<0.05, by Fisher’s transformation (Zr) of the cross correlation-coefficients and standard errors of Zr [21].

ARIMA is based on the assumption that the outcome series is stationary, which means that the mean and variances of the series are independent of time. To reduce the variances of the HPAI time series we took the log-transformed values. Autocorrelation (AC) and partial autocorrelation (PAC) functions were then examined to determine the initial AR order and MA order. First, we developed a univariable ARIMA model, where the response series depends only on its past values, followed by multivariable ARIMA models with the meteorological variables as covariates. Similarly, multiplicative univariable SARIMA model was developed, with only the outcome series at first, followed by the multivariable ones incorporating the meteorological parameters. For both kinds of multivariable models, meteorological parameters were first included one at a time, then two, three and so on. In this study, ARIMA and SARIMA models that included the meteorological variables were referred to as ARIMAX and SARIMAX, respectively. Lags in months were considered in both ARIMAX and SARIMAX models. Models with regression coefficients (β) with P<0.05 were included in this study to compare their performances. The performances of the models were compared based on Akaike’s Information Criterion (AIC) and error % of β.

All modeling and their statistical tests were performed using STATA software, version 11.0 (Stata Corporation, College Station, Texas, USA).

## Results

**Figure 1:**
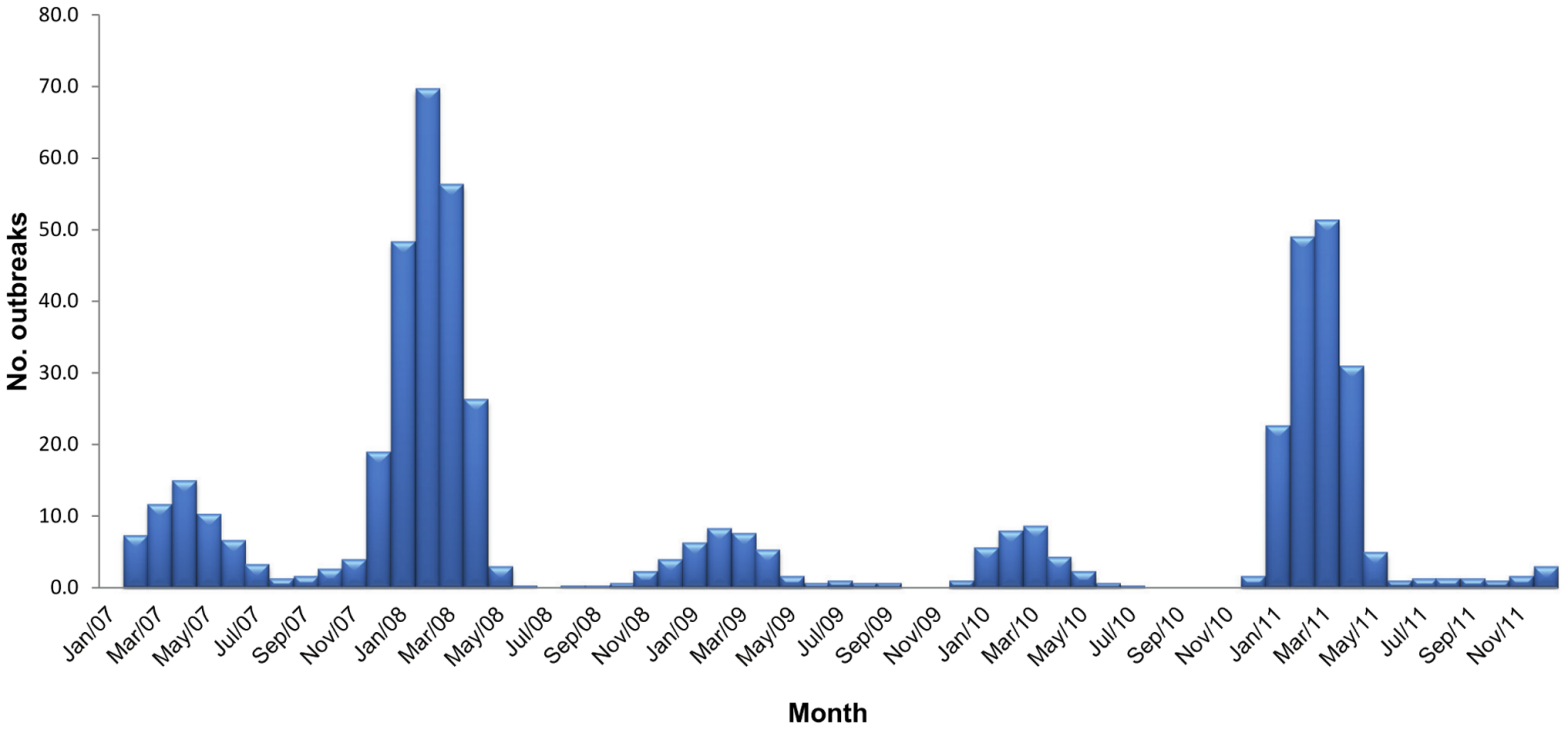
Three-monthly rolling average of highly pathogenic avian influenza (H5N1) outbreaks in Bangladesh in 2007–2011.

Based on 3-month rolling averages, the temporal trend of HPAI outbreaks from 2007 to 2011 is displayed in Figure 1. The numbers of outbreaks reported in 2007, 2008, 2009, 2010 and 2011 were 69, 226, 32, 31 and 171, respectively. The highest monthly number of outbreaks was 91, in February 2008, and the second highest was 79 in March 2011. There was a peak of outbreaks each year in the cooler months, December to February, extending to April in 2008 and 2011, and May in 2007. Bangladesh has 64 administrative districts. Spatially, 21 (32.8%), 46 (71.9%), 16 (25%), 13 (20.3%) and 37 (57.8%) districts, respectively, were recorded with HPAI outbreaks in the year 2007, 2008, 2009, 2010 and 2011 having the median numbers of outbreaks 2 (range 1–14), 3 (range 1–20), 1 (range 1–7), 1 (range 1–10) and 3 (range 1–28).

**Figure 2:**
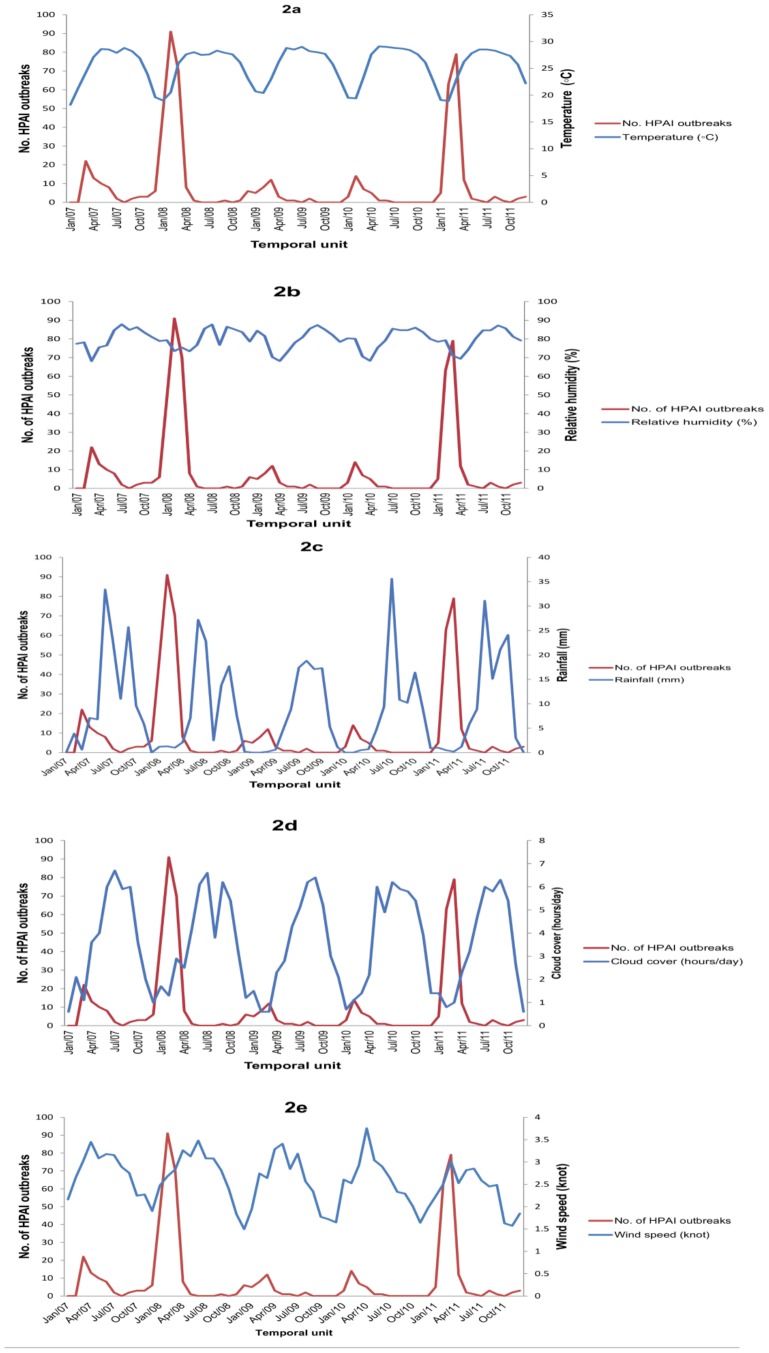
(a–e). Time series of monthly outbreaks of highly pathogenic avian influenza (HPAI) H5N1 and monthly mean average meteorological variables: (a) Temperature (°C), (b) Relative humidity (%), (c) Rainfall (in mm), (d) Cloud cover (in hour), and (e) Wind speed (knots), 2007–2011, Bangladesh.

Monthly means of average values of different meteorological factors and corresponding monthly numbers of HPAI outbreaks are displayed in Figure 2 (a–e).

There were significant correlations (p<0.05) between the outcome series and cloud cover (in hour), relative humidity (%), rainfall (in mm) and temperature (°C) at lag 0, 1 and 2 and wind speed (knots) at lag 2 (Table 1).

**Table 1:** Cross-correlation between meteorological variables and outbreaks of highly pathogenic avian influenza (HPAI) H5N1 in Bangladesh, 2007–2011.

Variable	Lag (in month)		
	0	1	2
Cloud cover (in hour)	−0.5758*	−0.6358*	−0.5544*
Relative humidity (%)	−0.5161*	−0.2528*	−0.0278*
Rainfall (in mm)	−0.4514*	−0.4549*	−0.4095*
Temperature (°C)	−0.5146*	−0.7646*	−0.7315*
Wind speed (knots)	0.1691	−0.1479	−0.4277*

*indicates significant at P<0.05.

For the first-order differenced series and the seasonally differenced series the AC and PAC approached to cutoff at lag 2. The ARIMA, ARIMAX, SARIMA and SARIMX models resulted as per the set criteria and the estimated coefficients are summarized in Table 2. Of the two univariable ARIMA models that resulted, ARIMA (1,0,1) had the best fit because of the lowest AIC (159.16) and error % (29.7%). Among the four SARIMA models resulted with the past seasonal inputs SARIMA (1,0,0) (0,1,1,12) has the best AIC. Inclusion of one or more meteorological variables as inputs did not improve the performance of any ARIMAX or SARIMAX models compared with the baseline univariable ARIMA and SARIMA models. However, there are ARIMAX and SARIMAX models of different orders with P<0.05. Among them ARIMAX (1,0,1) with RH and ARIMAX (1,0,1) with CC have the best fit and closest performances because of a similar AIC (∼150) and error % (36.5%). The model ARIMAX (1,0,1) with TE input series also fitted with the set P value, but had AIC of 154.41. Only one ARIMAX model, ARIMA (1,0,1) with two covariates – TE and RH fitted with the set p value while considering the MA order, but none of the ARIMAX models fitted with >2 covariates. None of the SARIMAX models with two or more meteorological inputs fitted. Noticeably, five SARIMAX models of different orders fitted with the RH input series. Of them SARIMAX (2,0,0) (1,0,0,12) and SARIMAX (1,0,1) (1,0,0,12) had a similar AIC. However, the latter has the best error %; consequently, we consider it the best SARIMAX model with RH. Two SARIMAX models - SARIMAX (1,0,0)(1,0,0,12) and SARIMAX (1,0,1) (1,0,0,12) also fitted with seasonal inputs of CC. On the basis of AIC the latter has a slightly better performance, although the error % was higher.

**Table 2:** Summary of model performances with the estimated coefficients for outbreaks of highly pathogenic avian influenza (HPAI) H5N1 associated with different meteorological variables, 2007–2011, Bangladesh.

Model	Fit		AR (β)	P	MA (β)	P	Meteorological Vars		
	AIC	Error%					Var	β	P
ARIMA (1,1,2)	159.18	35.3	0.5182	0.005	0.5045	0.063			
ARIMA (1,0,1)	159.16	**29.7**	0.5042	0.001	0.5054	0.001			
SARIMA (1,0,0)(0,1,0,12)	138.56	**10.2**	0.7442	<0.001	–	–			
SARIMA (1,0,0) (0,1,1,12)	131.36	**10.6**	0.6883	<0.001	−0.6112	0.004			
SARIMA (1,0,0) (1,0,0,12)	161.25	**18.7**	0.4609	0.001	–	–			
SARIMA (2,0,0) (1,0,0,12)	152.05	44.6	0.3180	0.025	–	–			
ARIMAX (1,0,1) with TE	154.41	40.8	0.3947	0.0105	0.5288	<0.001	TE	−0.1616	0.014
ARIMAX (2,1,0) with RH	164.28	45.5	0.2990	0.028	–	–	RH	−0.0691	0.003
			−0.3467	0.034					
ARIMAX (1,0,1) With RH	152.38	36.5	0.3980	0.006	0.5814	<0.001	RH	−0.0739	<0.001
ARIMAX (1,0,1) With CC	149.29	37.0	0.3841	0.007	0.5566	<0.001	CC	−0.2645	0.002
ARIMAX (1,0,1) With TE and RH	147.21	35.23	0.3124	0.066	0.5770	<0.001	TE	−0.1481	0.007
							RH	−0.0714	0.002
SARIMAX (S) (2,0,0) (1,0,0,12) with RH	148.21	47.1	0.2986	0.034	–	–	RH	−0.0613	0.005
SARIMAX (S) (1,0,0) (1,0,0,12) with RH	157.35	33.1	0.4235	0.003	–	–	RH	−0.0716	0.008
SARIMAX (S) (1,0,0) (0,0,1,12) with RH	158.98	**21.0**	0.6557	<0.001	0.3872	0.005	RH	−0.0784	0.004
SARIMAX (S) (1,0,1) (1,0,0,12) with RH	149.47	39.5	0.3225	0.011	0.5639	<0.001	RH	−0.0697	0.002
SARIMAX (S) (1,0,1) (0,0,1,12) with RH	150.37	39.6	0.3806	0.012	0.2769	0.051	RH	−0.0738	0.001
SARIMAX(S) (1,0,0)(1,0,0,12) with CC	157.46	44.8	0.3404	0.025	–	–	CC	−0.0229	0.043
SARIMAX(S) (1,0,1) (1,0,0,12) with CC	149.09	51.0	0.2429	0.050	–	–	CC	−0.2263	0.011

Abbreviations: ARIMA = Autoregressive Integrated Moving Average; S = Seasonal (Multiplicative); X = with Meteorological Input Series; AIC = Akaike’s Information Criterion; AR = Autoregressive; β = Estimated coefficient; MA = Moving Average; Var = Variable; TE = Average Air Temperature (°C); RH = Relative Humidity (%); CC = Average Cloud Cover (in hour).

## Discussion

The relationship between the meteorological factors and the occurrence of HPAI outbreaks was assessed using 5 yearly data from Bangladesh, which is severely affected by HPAI. Initially, ARIMA and SARIMA models of different orders with first-order series were tested. ARIMA (1,0,1) was the best non-seasonal univariable model, where HPAI outbreaks depend on the outbreaks in the previous month. The best two multiplicative seasonal univariable models, where the past outcome series are the inputs, were SARIMA (1,0,0) (0,1,1,12) and SARIMA (1,0,0) (1,0,0,12), indicating non-dependency and dependency on the outbreaks in the previous season (i.e. 1 year). Because these SARIMA models have a better AIC (Table 2) compared with ARIMA (1,0,1), it can be assumed that HPAI outbreaks are modeled best when multiplicative seasonality is incorporated.

Yearly temporal patterns of HPAI outbreaks in Bangladesh varied in magnitude and spatial distributions, although there was a peak of outbreaks each year in the cooler months. Because 32.8%, 71.9%, 25%, 20.3% and 57.8% of the total districts across the country were recorded with HPAI outbreaks, respectively, in the year 2007, 2008, 2009, 2010 and 2011 we used the aggregated data of the climatological variables recorded from all the 35 observatory stations located in the country.

The evolutionary history of the virus based on phylogenetic analysis of the HA gene of representative isolates from HPAI outbreaks in Bangladesh revealed that only the clade 2.2 of the virus subtype H5N1 caused the outbreaks from 2007 to 2010 [22]. This was also the predominant clade identified from the outbreaks in 2011. However, two new clades: 2.3.2 and 2.3.4 were also associated with some outbreaks, suggesting their new introductions to the country in the year 2011 along with the existing circulation of clade 2.2 since 2007 [23].

The incorporation of one or any combination of the meteorological parameters as inputs in the SARIMAX models did not improve the performance of any model compared with the corresponding univariable model. There is only one multivariable model: ARIMAX (1,0,1) with inputs of two meteorological variables: RH and TE has p<0.05 for the estimated coefficients of MA, suggesting evidence against the null hypothesis [21] that no association exists between the occurrence of HPAI and RH along with TE. In single inclusion of RH with different AR and MA orders, there are 2 ARIMAX and 5 SARIMAX models where RH shows significant association with the outcome series (P<0.05). Among these are three very closely performing SARIMAX models because of similar AIC, ranging from 148 to 150; two of which: SARIMAX (S) (2,0,0) (1,0,0,12) and SARIMAX (S) (1,0,1) (1,0,0,12) depend on the outbreaks in the previous 12 months, but the third one, SARIMAX (S) (1,0,1) (0,0,1,12) does not. Two SARIMAX models where estimated CC coefficient is significant (p<0.05) also depend on the HPAI outbreaks in the past 12 months. The best SARIMAX models, especially those with RH covariate, indicate that RH is probably the most important meteorological predictor of the seasonal trend of HPAI outbreaks in Bangladesh. The estimated coefficients for the outcome and RH of these models illustrate that monthly decreasing in RH is associated with monthly increasing numbers of HPAI outbreaks: this association has been observed over the last five years in Bangladesh since 2007.

The contributory role of TE on seasonal HPAI outbreaks in Bangladesh is difficult to explain from this study because no SARIMAX model has it as a significant covariate. But in a particular year TE alone or with RH might have some influence, as evidenced in ARIMAX (1,0,1) with TE and ARIMAX (1,0,1) with TE and RH (Table 2), where monthly decreasing in TE is associated with monthly increasing number of HPAI outbreaks. However, decreasing monthly CC might be associated with decreasing HPAI outbreaks in a year and the previous one. The models wherein RH is a significant predictor are more diverse in AR and MA orders than those with CC as a significant covariate.

To our knowledge, this is the first report on the roles of RH in seasonal occurrence of HPAI outbreaks. However, RH along with rainfall and land surface temperature was a significant predictor for human influenza in Hong Kong [17]. RH and TE are often associated with human influenza in Tokyo and in temperate regions [19]. Mahamat et al [24] reported that an increase of 1 g/kg of specific humidity (SH) resulted in a decrease of 11% in influenza-like illness incidence in French Guiana. To calculate SH, daily average of surface pressure is also required along with daily records of TE and RH. Because we had no daily surface pressure data we used RH, instead of SH in our analysis, as applied by many similar time-series studies [17], [25]. Dry air with low RH and low temperature during the winter months seems to increase the transmission of HPAI H5N1 virus among poultry. Dry air might favor the transmission of HPAI H5N1 through aerosol, and its longer survival in dry air, although the present study has no evidence to verify this. However, Lowen et al [26] found that at low RH and temperature, transmission of human influenza virus is most efficient. The lowest monthly mean average RH was ∼70% in the driest months, in winter, and might reach as high as ∼ 88% in the monsoon in Bangladesh, as seen in the 5 years’ data used in this study. What triggers the first outbreak in a year is unknown, but the virus introduction by migratory birds is plausible [16], [27]. However, the outbreaks peak in the dry months when the RH is ∼70%, and almost cease when the monsoon gradually takes over with RH as high as 88%. Thus moist heat might play an important role in inactivating the virus in the air, resulting in a reduced rate of virus transmission. RH (not rainfall) seems to be an important parameter used in predicting HPAI trend in Bangladesh.

Decreasing CC is linked to decreasing RH, and possibly to decreasing TE, because radiation of heat from earth surface is greater when the sky is cloud-free. There were no models where all these three could be fit as significant covariates.

Bangladesh still predominantly relies on passive surveillance for HPAI. This passive reporting depends very much on monetary compensation. In its absence, farmers can sell birds incubating the virus in live markets, helping spread the virus. Stopping of providing monetary compensation could be the reason why HPAI reported cases are surprisingly very low from 2013 onwards in Bangladesh. The virus is still very active in Bangladesh. In response to farmers’ demand, the country has introduced vaccination against the virus in two districts [28]. Controlling HPAI by vaccination is a debated issue, and the importance of vaccination must not substitute for good biosecurity.

The best models from this study might also be applicable for predicting HPAI in other countries, particularly in South- and South-East Asia, because of similar climate.

In conclusion, SARIMA (1,0,0) (0,1,1,12) and SARIMA (1,0,0) (1,0,0,12) are the two best models and their performances are better than any ARIMA, ARIMAX or SARIMAX models, indicating a seasonal trend of HPAI outbreaks in Bangladesh. RH is a significant predictor in two ARIMAX and five SARIMAX models with different AR and MA orders, and the estimated coefficients reveal that decreasing monthly RH alone or along with decreasing monthly TE might be associated with increasing monthly number of HPAI outbreaks. On its own, CC is also a significant covariate in two SARIMAX models.
